# Editorial Comment from Dr Bilim to Testicular sarcoidosis with bilateral scrotal swelling

**DOI:** 10.1002/iju5.12131

**Published:** 2019-11-12

**Authors:** Vladimir Bilim

**Affiliations:** ^1^ Kameda Daiichi Hospital Niigata Japan

Sarcoidosis is a rare disease of unknown pathogenesis. Urogenital sarcoidosis is very rare. PubMed search for “testicular sarcoidosis” returned only 26 articles. It usually affects the epididymis, followed by testis and vas deferens. It typically occurs in young adults. Symptoms are usually asymptomatic unilateral or bilateral scrotal swelling, testicular pain. Testicular sarcoidosis could be diagnosed in patients whose chief complaint is infertility. In the article by Kimura *et al*.,^1^ the patient presented with bilateral scrotal swelling.

The differential diagnosis of urogenital sarcoidosis includes testicular tuberculosis, malignant lymphoma and testicular tumor.^2^

Diagnosis routine usually involves ultrasound examination of scrotum. Gallium‐67 scintigraphy also can be done. Sonographic examination of the scrotum usually reveals multifocal well‐defined, round, hypoechoic intratesticular lesions.^3^, ^4^ A testicular biopsy should be taken to confirm the diagnosis of sarcoidosis and exclude malignancy. If a patient has no previous history of sarcoidosis, chest X‐ray or computed tomography scan, to confirm lung sarcoidosis, and serum markers panel (angiotensin‐converting enzyme, KL‐6 and sIL‐2R) are performed. In the presented case, the patient has been diagnosed with lung sarcoidosis 2 years prior to presentation to the urologists.

The association between infertility and sarcoidosis is unknown. There are several reports on azoospermia and oligozoospermia in patients with sarcoidosis. Several causative explanations are possible. First is replacement of testicular tissue with granulomas. Another is sarcoidosis of epididymis resulting in obstructive azoospermia. Local effect of granuloma on spermatogenesis or systemic manifestation of sarcoidosis as secondary hypogonadism might also happen. Steroid treatment alleviating symptoms of sarcoidosis usually result in improvements in sperm count.^5^ The patients received prednisolone in a dose 40 mg/day, which was tapered to maintenance dose of 2.5 mg/day. Lung lesions demonstrated radiologic improvement. However, sperm count showed only mild improvement (7 million/mL). Increase in the prednisolone dose might result in further improvement of sperm count. Otherwise, the patient can opt for assisted reproductive technology.

Chronic sarcoidosis tends to relapse. Thus, exacerbation of symptoms in patients with testicular sarcoidosis could happen after remission period.

## Conflict of interest

The author declares no conflict of interest.
